# Competitive ability underpins the effect of spatial aggregation on plant performance

**DOI:** 10.1002/ecy.70075

**Published:** 2025-04-10

**Authors:** Naoto Shinohara, Haruna Ohsaki

**Affiliations:** ^1^ Center for Ecological Research Kyoto University Otsu Shiga Japan; ^2^ Department of Biological Sciences Tokyo Metropolitan University Hachioji Tokyo Japan

**Keywords:** aggregation, coexistence, competition, competitive ability differences, niche differences, plants, spatial structure

## Abstract

Most plant species exhibit spatially clustered distributions. Theory suggests such conspecific aggregation can delay competitive exclusion by sparing weak competitors. However, the extent to which spatial aggregation increases species performance and which species are likely to benefit from it remain largely unknown. In this study, we asked (1) whether spatial aggregation enhances plant performance and (2) whether the effects are biologically predictable. For the second question, we focused on “the competition‐relatedness hypothesis” and the “competitive asymmetry hypothesis,” which relate the effect of spatial arrangement to niche and competitive ability differences between species, respectively. We performed phylogenetic meta‐analyses to investigate whether phylogenetic and ecological differences among competitors explain the effect of spatial arrangement. We found idiosyncratic responses of plant species to spatial aggregation. While some species performed better when conspecific individuals were aggregated, others did so when conspecifics and heterospecifics were randomly distributed. The non‐negligible number of species benefiting more from intraspecific aggregation indicates that intraspecific competition is sometimes weaker than interspecific competition. Further, the result contrasts with the assumption of the competition‐relatedness hypothesis, which postulates the strongest competition among conspecifics, suggesting that this hypothesis does not hold for at least these species. Although phylogeny did not predict the effect of spatial arrangement, interspecific plant height differences did: Species performed better in an aggregation treatment when they were smaller than competitors. Collectively, our results lend more support for the competitive asymmetry hypothesis that interspecific differences in competitive ability underpin the effect of spatial arrangement on plant performance. Moreover, they suggest that spatial processes, such as dispersal limitation, may play an important role in plant coexistence.

## INTRODUCTION

**FIGURE 1:**
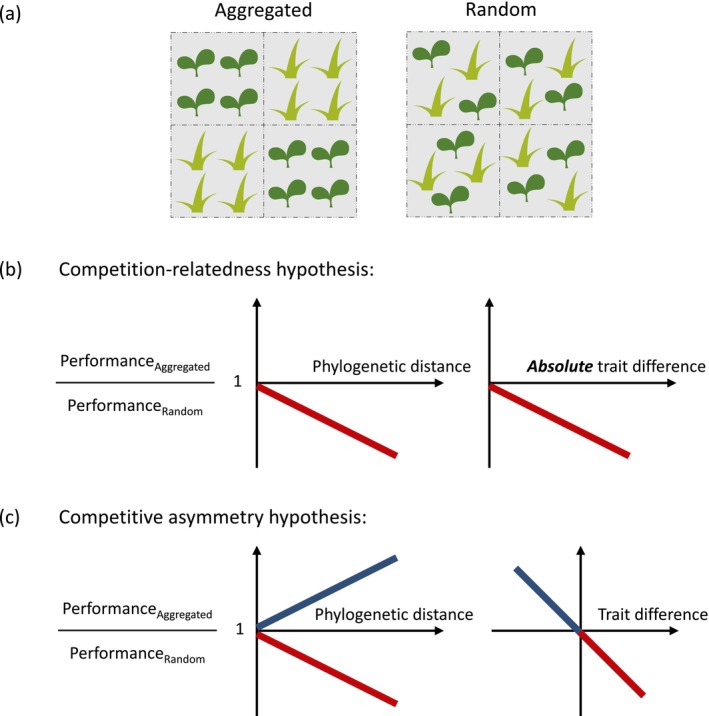
(a) A schematic representation of experiments designed to measure plant performances in different spatial arrangements (aggregated vs. random). (b) Predictions of the competition‐relatedness hypothesis and (c) the competitive asymmetry hypothesis concerning the correlation between the ratios of plant performance in two spatial arrangements and the phylogenetic distances between competitors (left) and ecological trait dissimilarities (right). The lines are colored blue and red, respectively, for data with positive and negative response ratios. Note that in panel (c) the *x*‐axis for interspecific trait differences represents the raw difference, whereas in panel (b) it represents the absolute difference. Plant illustrations in panel (a) were created by Haruna Ohsaki.

In natural ecosystems, species distributions are rarely random. Instead, organisms across a wide range of taxonomic groups typically exhibit spatially aggregated distributions due to, for instance, dispersal limitation (Condit et al., 2002; Karlson et al., 2007). One of the key consequences of this nonrandom spatial arrangement is its association with community dynamics and species diversity (Atkinson & Shorrocks, 1981; Murrell et al., 2001). Conspecific aggregation makes intraspecific interactions occur more frequently than interspecific interactions, thereby delaying competitive exclusion and potentially promoting species coexistence (Chesson & Neuhauser, 2002; Murrell et al., 2001). Therefore, previous studies have rigorously compared the performance (e.g., biomass, fecundity, and survival) of species under conditions of conspecific aggregation versus random arrangement of conspecific and heterospecific individuals (Figure 1a). They consistently show that, whether in terrestrial ecosystems (e.g., plants [Stoll & Prati, 2001; Monzeglio & Stoll, 2005], insects [Fader & Juliano, 2013; Kadowaki & Inouye, 2015]) or marine ones (e.g., corals [Idjadi & Karlson, 2007], benthic invertebrates [Hart & Marshall, 2009]), the effects of spatial arrangement are species‐specific—some species exhibit decreased performance when aggregated, while others benefit from aggregation. Certain biological characteristics may drive these idiosyncratic responses, but our understanding of such underpinnings is lacking.

Some evidence suggests that the effect of spatial arrangement on species performance is predictable from ecological traits or phylogenetic information. This is because species' responses to spatial arrangement (i.e., aggregated vs. random) naturally reflect the relative strength of intraspecific and interspecific competition that the focal species experience. Species suffering more from conspecific aggregation (or random arrangement) are more susceptible to intraspecific (interspecific) competition. As such, biological underpinnings of the effect of spatial arrangement can be understood from two viewpoints, niche differences and competitive ability differences between species, which we relate to the “competition‐relatedness hypothesis” and “competitive asymmetry hypothesis,” respectively (Mayfield & Levine, 2010).

First, the difference in a species' responses to conspecific and heterospecific neighbors could reflect its niche difference from the competitors (Chesson, 2000). The more dissimilar a focal species' niche is from its competitors, the better it should perform when surrounded by heterospecific (in addition to conspecific) individuals in a random arrangement, due to smaller niche overlap. Conversely, species may perform worst in an aggregation treatment due to the greater niche overlap with conspecific neighbors. Previous studies have implied that interspecific niche differences are sometimes predictable from their differences in ecological traits (Kraft et al., 2015; Pérez‐Ramos et al., 2019) or phylogenetic distance (Germain et al., 2016; Violle et al., 2011, but see Godoy et al., 2014; Narwani et al., 2013). In addition, in plant communities, for instance, evidence indicates that pairs of more closely related species tended to experience more negative plant–soil feedback (Crawford et al., 2019) or negative density‐dependent mortality (Zhu et al., 2015), both of which suggest that phylogenetically more distant species differ more in their niches. Therefore, one may expect that with increasing absolute trait differences or phylogenetic dissimilarity from competitors, species increasingly perform better in a random treatment due to smaller niche overlaps (Figure 1b). Notably, this hypothesis does predict that species performance in an aggregation treatment is always lower than that in a random treatment, since intraspecific competition is assumed to be the strongest due to greater niche overlaps (Figure 1b).

The second hypothesis, which we refer to as the competition‐asymmetry hypothesis, posits that there is a hierarchy in species' competitive effects on other individuals, and these effects are determined by certain traits or are phylogenetically conserved (Godoy et al., 2014; Mayfield & Levine, 2010). To illustrate this hypothesis and its relevance to the effects of spatial arrangement, let us consider two species with different per capita effects on other individuals: stronger affecter and weaker affecter species. The stronger affecter would perform worse in an aggregation treatment (due to stronger intraspecific competition) than when surrounded by heterospecifics in a random treatment (exposed more to interspecific competition from weaker affecters). Conversely, the weaker affecter would decrease its performance when randomly arranged, because it interacts more frequently with heterospecifics with stronger effects. Therefore, responses to spatial arrangement could reflect species' relative competitive abilities. Certain ecological traits are known to underpin competitive abilities (e.g., in plant communities, plant height [Ferenc & Sheppard, 2020; Gaudet & Keddy, 1988] and seed size [Turnbull et al., 2004]), which thereby potentially determine species' responses to spatial arrangement. If such traits are phylogenetically conserved, we expect that competitive ability differences, and hence responses to spatial arrangement, are phylogenetically structured (Germain et al., 2016; Godoy et al., 2014, but see Narwani et al., 2013). Accordingly, this hypothesis predicts that a focal species' performance in an aggregated treatment, compared with that in a random treatment, is better when its trait value associated with competitive ability is lower than that of the competitor or when the focal and competitor species are phylogenetically more distant (Figure 1c). Note that this hypothesis predicts that the response ratio can be either higher or lower than 1, depending on whether a focal species is a weaker or stronger affecter (Figure 1c).

To understand the effect of spatial arrangement on species performance and its biological underpinnings, we synthesized previous experimental studies and performed a phylogenetic meta‐analysis. We focused exclusively on experiments conducted in plant communities for their simple life history characterized by being sessile and living in permanent habitats. Still, we argue that the findings from plants could apply to more dynamic systems where spatial arrangements play a critical role in species coexistence (e.g., insects using ephemeral resources [Fader & Juliano, 2013; Kadowaki & Inouye, 2015] and marine sessile organisms [Hart & Marshall, 2009; Idjadi & Karlson, 2007]). We first asked (1) whether species perform better or worse when spatially aggregated than when randomly arranged. We predicted that spatial aggregation decreases species per capita performance due to stronger intraspecific competition than interspecific competition as suggested by a recent meta‐analysis (Adler et al., 2018). Then, we tested (2) the correlation between the ratio of species' performance in aggregation and random treatments, and phylogenetic and ecological dissimilarities between focal and competitor species, as predicted from the competition‐relatedness and competitive asymmetry hypotheses (Figure 1b,c). While the competition‐relatedness and competitive asymmetry hypotheses yield similar predictions, they differ in that the former predicts only negative response ratios (Figure 1b), whereas the latter predicts both positive and negative values (Figure 1c). Additionally, the competition‐relatedness hypothesis predicts that absolute trait differences correlate with the response ratio (Figure 1b), whereas the competitive asymmetry hypothesis suggests that raw trait differences are the key correlate (Figure 1c).

## MATERIALS AND METHODS

### Data extraction

To obtain relevant studies, we conducted a keyword search using the Web of Science (www.webofknowledge.com/WOS, accessed on June 20, 2023). We looked for experimental studies that measured the performance of plants in multiple spatial arrangement treatments (e.g., random vs. aggregated, Figure 1a). Our search terms included (plant* OR vegetation*) AND (experiment* OR manipulat* OR cultivat*) AND (random* OR even OR dispers* OR segregate*) AND (aggregat* OR cluster*) AND (spa* OR distribut* OR arrange*) in their title, abstract, or authors' keywords (asterisks are wildcards). This search identified 994 papers; we reviewed their titles and abstracts to judge their relevance to our aim, resulting in 50 papers potentially contributing to our analysis. We then read through the main texts and extracted information essential for the meta‐analysis: (1) measures of the performance (e.g., biomass) of focal species in the two spatial arrangement treatments (i.e., random vs. aggregated distribution), (2) variance in the performance measures (SD or SE), (3) number of replicates per treatment, and (4) identity of the focal and competitor species. We found 11 papers containing sufficient information (Cai et al., 2017; Dong et al., 2013; Houseman, 2014; Lamošová et al., 2010; Liao et al., 2014; McKenna & Yurkonis, 2016; Monzeglio & Stoll, 2005, 2008; Porensky et al., 2012; Stoll & Prati, 2001; Wassmuth et al., 2009). We added one paper to the list that did not appear in our search but was known to us beforehand (Xue et al., 2018). When available, we also extracted additional information from the papers: density of individuals (number of planted individuals or sown seeds per square meter), duration of the experiment, and size of the unit of aggregation (size of grids in Figure 1a). All the focal species in these studies happened to be herbaceous.

Some studies reported multiple performance measures for single observations, such as total aboveground biomass, belowground biomass, and the number of individuals. In such cases, we used aboveground biomass and discarded the other measures, since it was the most frequent performance measure. For studies that did not measure aboveground biomass, we retrieved the most relevant performance measure, such as percent coverage. When a single study measured performance multiple times for the same samples, we retrieved values measured at the last time. Consequently, we obtained 89 independent observations from 12 studies (most studies used multiple focal species).

As far as we could tell from the papers, the experiments were conducted in presumably nutrient‐rich conditions (e.g., plots are located in former agricultural areas [Houseman, 2014; McKenna & Yurkonis, 2016] or fertilized [Monzeglio & Stoll, 2008; Stoll & Prati, 2001]), and plants were watered sufficiently (e.g., Monzeglio & Stoll, 2005; Stoll & Prati, 2001). Accordingly, we have no reason to suppose the existence of significant environmental heterogeneity among studies that may influence plant performance (for instance, increased performance in conspecific aggregation in harsh environments). Our meta‐regression also indicated that among‐study variation in the effect size was ignorable compared with interspecific variation (Appendix S1: Table S1, discussed later). Also, no environmental differences between random and aggregated treatments are expected as their plots were typically located in adjustment to each other.

### Phylogenetic information

In our dataset, 53 plant species were used as focal species (whose performance was measured), while 58 were used as competitors, resulting in 60 species appearing as either focal or competitor species. We obtained a phylogenetic tree for these species using the “rtrees” package (Li, 2023) in R (R Core Team, 2024) based on a mega‐tree that was primarily derived from Smith and Brown (2018) and Jin and Qian (2019). Before this, we standardized species names according to the World Flora Online using the “WorldFlora” package (Kindt, 2020). Using this phylogenetic tree, we calculated a phylogenetic correlation matrix based on branch lengths between species, using the “vcv” function in the “ape” package (Paradis & Schliep, 2019) with an assumption of the Brownian model of evolution.

### Ecological traits

For each species, we obtained ecological traits potentially representative of interspecific niche or fitness differences (and thus associated with the response to spatial arrangement): plant height (in meters) and seed mass (in milligrams). We retrieved each species' representative value, which was calculated as an average of multiple samples, from publicly available databases (Database of European Vegetation, Habitats and Flora [www.floraveg.eu] and TRY [Kattge et al., 2020, www.try-db.org]), or previous studies (Appendix S1: Table S2). We additionally recorded each species' functional type (grass, forb, or legume) and life history (annual or perennial) to account for the possibility that these traits are associated with species' niche and competitive ability. Finally, as a trait that could be a cause of aggregated distributions, we collected species' dispersal modes. They were determined by assessing prior studies or judged from seed images from reliable databases (POWO, 2024; GBIF, 2024): Seeds with wings or pappus were classified as wind‐dispersal (anemochory), while seeds without any dispersal‐related characteristics were nonspecialized, gravity‐dispersal. Other dispersal modes (including animal‐dispersal [endozoochory]) were grouped into one category due to the limited number of such cases. These trait data were available for 70%–80% of the species (Appendix S1: Table S2).

### Phylogenetic meta‐analysis

We constructed phylogenetic meta‐analysis models (Cinar et al., 2022; Koricheva et al., 2013; Nakagawa & Santos, 2012) to test our two predictions. In the models, the response variable was the effect size of the treatments: the natural log of the response ratio $\ln\left(\frac{y_{\text{Aggregated}}}{y_{\text{Random}}}\right),$ where *y*_Aggregated_ and *y*_Random_ are the performance measures in the two treatments. A ratio larger (or smaller) than 1 indicates that the performance of a species is higher (or lower) when conspecifics are aggregated than when randomly arranged.

First, to study the overall mean effect size, we considered the following model:

(1) $$\ln{\left(\frac{y_{\text{Aggregated}}}{y_{\text{Random}}}\right)}_{i}=\mu +u_{j\left[i\right]}+v_{k\left[i\right]}+a_{k\left[i\right]}+m_{i},$$

$$\begin{array}{l} u_{j}\sim N\left(0,{\sigma_{u}}^{2}\right),v_{k}\sim N\left(0,{\sigma_{k}}^{2}\right),a_{k}\sim N\left(0,{\sigma_{a}}^{2}A\right),\\ \;m_{i}\sim N\left(0,{\sigma_{i}}^{2}\right), \end{array}$$

where *i*, *j*, and *k* stand for observation, study, and species identity, respectively. The parameter of interest concerning our first hypothesis was μ, the meta‐analytic mean of the effect size. We adopted a so‐called “random‐effect model” where the true effect size was assumed to differ among studies by incorporating a study‐specific random effect *u*_*j*_ with the among‐study variance being σ_*u*_^2^. Also, we considered among‐species heterogeneity in the effect size by introducing *v*_*k*_ and *a*_*k*_, which respectively correspond to non‐phylogenetic and phylogenetic species‐level random effects. The non‐phylogenetic species‐level random effect, *v*_*k*_, was assumed to follow a normal distribution with variance σ_*k*_^2^. The phylogenetic species‐level random effect, *a*_*k*_, was assumed to follow a multivariate normal distribution with mean 0 and variance–covariance matrix σ_*a*_^2^ *A*, where *A* is the phylogenetic correlation matrix obtained from the mega‐tree. We considered these two species‐level random effects separately, since ignoring non‐phylogenetic random effects may underestimate variance in effect size and thus increase the risk of Type II error (Cinar et al., 2022). The last term, *m*_*i*_, represents the sampling error of each observation, which was assumed to be normally distributed around 0 with corresponding variance σ^2^_*i*_. The observation‐specific variance σ^2^_*i*_ was approximated as ${\sigma^{2}}_{i}=\frac{{{\mathrm{SD}}_{\text{Aggregated}}}^{2}}{n_{\text{Aggregated}}\times {y_{\text{Aggregated}}}^{2}}+\frac{{{\mathrm{SD}}_{\text{Random}}}^{2}}{n_{\text{Random}}\times {y_{\text{Random}}}^{2}}$, where *n* and SD are the number of replicates and the standard deviation of performance measure values for each treatments, respectively (Koricheva et al., 2013). The parameters were estimated using the “rma.mv” function in the “metafor” package (Viechtbauer, 2010) via the restricted maximum likelihood estimation.

To test our second prediction (the correlation between the response ratio and phylogenetic or trait dissimilarities between competitors, Figure 1b), we first calculated the phylogenetic distance between focal (whose performance was measured) and competitor species. Note that in our dataset, some experiments used more than one species as competitors (7 species in median and 15 in maximum); therefore, we calculated the average phylogenetic correlation between focal species and their multiple competitors. This might blur the effect of phylogenetic distance, a potential limitation of our approach. We included the average phylogenetic distance, phylo.dist_*i*_ (1 − the average phylogenetic correlation) as the explanatory variable in the regression model as

(2.1) $$\ln{\left(\frac{y_{\text{Aggregated}}}{y_{\text{Random}}}\right)}_{i+}=\mu +\beta_{\text{phylo},+}\times \text{phylo}.{\text{dist}}_{i}+u_{j\left[i\right]}+v_{k\left[i\right]}+a_{k\left[i\right]}+m_{i},$$

(2.2) $$\ln{\left(\frac{y_{\text{Aggregated}}}{y_{\text{Random}}}\right)}_{i-}=\mu +\beta_{\text{phylo},-}\times \text{phylo}.{\text{dist}}_{i}+u_{j\left[i\right]}+v_{k\left[i\right]}+a_{k\left[i\right]}+m_{i}.$$

These models are the same as model 1 except for an additional term phylo.dist_*i*_. Since we predicted that phylogenetic distance influences the absolute values of the response ratio (Figure 1b), the models were separately fitted to two datasets that contain only positive (model 2.1) or negative (model 2.2) response ratios. According to our prediction (Figure 1b), we expected β_phylo,+_ > 0 and β_phylo,–_ < 0.

To test the effect of ecological trait differences, we constructed models similar to model 2, where the explanatory variable phylo.dist_*i*_ was replaced by a difference in plant height (height.diff_*i*_) or seed size (seed.diff_*i*_) between a focal species and the average of its competitors (focal − competitor average):

(3.1) $$\ln{\left(\frac{y_{\text{Aggregated}}}{y_{\text{Random}}}\right)}_{i}=\mu +\beta_{\text{height}}\times \text{height}.{\text{diff}}_{i}+u_{j\left[i\right]}+a_{k\left[i\right]}+m_{i},$$

(3.2) $$\ln{\left(\frac{y_{\text{Aggregated}}}{y_{\text{Random}}}\right)}_{i}=\mu +\beta_{\text{seed}}\times \text{seed}.{\text{diff}}_{i}+u_{j\left[i\right]}+a_{k\left[i\right]}+m_{i}.$$

We dropped the non‐phylogenetic species‐level random effect *v*_*k*_, assuming that these ecological traits explain the species‐specificity in the response ratios. We expected β_height_ <0 and β_seed_ <0 because plant height and seed size would represent competitive abilities of plants (Ferenc & Sheppard, 2020; Gaudet & Keddy, 1988; Turnbull et al., 2004), and thus, we expected that the larger these trait values of a focal species are, the worse it performs when spatially aggregated. These models were fitted to datasets where the trait values were available for focal species (*N* = 70 for the plant height model and *N* = 68 for the seed size model). In cases where trait values were unavailable for some competitor species, we excluded those species from the calculation of competitor averages. Additionally, to test the prediction of the competition‐relatedness hypothesis (Figure 1b), we fit similar models where the explanatory variables in model 3 were replaced by their absolute values (i.e., absolute differences in plant height or seed size) to the data with negative response ratios.

For the categorical trait data (seed dispersal modes [wind, gravity, or others], functional types [grass, forb, or legume], and life history [annual or perennial]), it was not straightforward to calculate their difference between focal and competitor species. Hence, to examine whether the response to spatial arrangement is associated with these traits, we developed another model:

(4) $$\begin{array}{c} \ln{\left(\frac{y_{\text{Aggregated}}}{y_{\text{Random}}}\right)}_{i+}=\mu +\beta_{\text{disp}}\times {\text{disp}}_{k\left[i\right]}+\beta_{\text{func}}\times {\text{func}}_{k\left[i\right]}\\ \;+\beta_{\text{life}}\times {\text{life}}_{k\left[i\right]}+u_{j\left[i\right]}++a_{k\left[i\right]}+m_{i}, \end{array}$$

where disp_*k[i]*_, func_*k[i]*_, and life_*k[i]*_ indicate the dispersal mode, functional type, and life history of species *k*, respectively. These explanatory variables were introduced as categorical, with “gravity” (for dispersal mode), “forb” (functional type), or “annual” (life history) set as a reference level for each variable.

We also examined the possible effects of experimental settings on the response ratio. Specifically, we expected that plant density and duration of the experiment increase the absolute effect of spatial arrangement on plant performances. To test this, we replaced the mean phylogenetic distance in model 2 by (1) the log‐transformed density of sown seed or planted individual (per square meter), (2) the duration of the experiment (in months), or (3) the log‐transformed size of the unit of aggregation (the size of grids in Figure 1a, in square meters). In these additional models, we did not consider the study‐specific random effect (*u*_*j*_) assuming that it is reflected in the experimental setting variables. Note that density measures differed among studies, with some reporting the number of planted individuals while others reporting the number of seeds sowed. We therefore modeled the effect of plant density separately for these two types of data. We found that experimental duration or plant density did not strongly affect the response ratio (Appendix S1: Figure S1a,b). In contrast, the response ratio deviated further from zero as increasing unit size of aggregation in the data with negative response ratios (Appendix S1: Figure S1c). This significant relationship is reasonable, as decreasing the unit size of aggregation makes a distribution closer to random.

All the calculations were performed using R (R Core Team, 2024) and figures were drawn using the “ggplot2” package (Wickham, 2016).

## RESULTS AND DISCUSSION

### The overall effect of spatial arrangement

**FIGURE 2:**
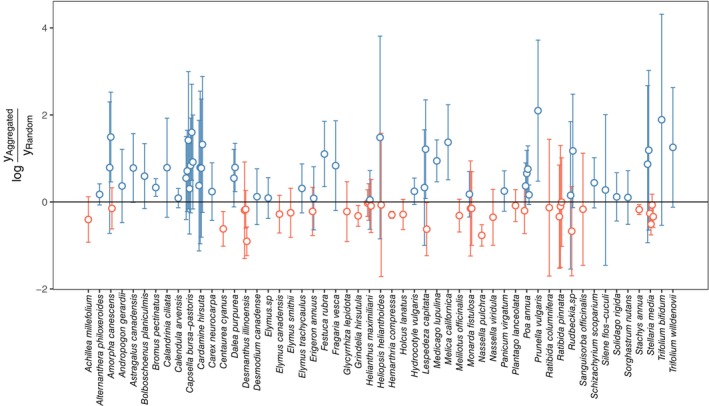
Observed species' response ratios to spatial arrangements (the log‐ratio of the performances in aggregated and random treatments). Each point represents a single observation with a bar representing its observation error (±1.96 × σ_*u*_). Points with positive and negative response ratios are colored with blue and red, respectively. For species with multiple observations, the points and lines were slightly shifted horizontally for visualization purposes.

Consistent with previous studies (e.g., Monzeglio & Stoll, 2005; Stoll & Prati, 2001), we found idiosyncratic patterns in plant responses to spatial arrangement. Some species perform better when conspecifics were aggregated (blue points in Figure 2), while others do so in random arrangements (red points in Figure 2). The overall response ratio, $\ln\left(\frac{y_{\text{Aggregated}}}{y_{\text{Random}}}\right)$, did not significantly deviate from zero (μ = 0.112 ± 0.082 [SE], *p* = 0.172).

This finding suggests that interspecific competition is sometimes comparable to intraspecific competition. This conclusion contrasts with a recent meta‐analysis showing that intraspecific competition was stronger (Adler et al., 2018). The inconsistency may be partly attributable to a difference in analytical approaches: whereas we used a random‐effect model that explicitly considers heterogeneity in the reliability of observations, the previous meta‐analysis weighted all data equally (due to the lack of necessary information for adopting random‐effect models), which might lead to the underestimation of the variance. Consistent with this interpretation, in Adler et al. (2018), the effect sizes were smaller (closer to neutral) for the manipulative (than observational) and greenhouse (than field) studies, where observation errors are presumably smaller. Another meta‐analysis incorporating the observation‐specific variance identified no significant difference between the degrees of intraspecific and interspecific competition in primary producers (Gurevitch et al., 1992). Therefore, we conclude that strong self‐regulation may not be widespread in plant communities.

Our observation of many species demonstrating positive response ratios (Figure 2) contrasts with a prediction of the competition‐relatedness hypothesis, which assumes the strongest competition among conspecifics due to greater niche overlap (Figure 1b). Instead, a non‐negligible number of species suffered more from interspecific competition than from intraspecific competition. This leads us to conclude that there is a hierarchy in competitive abilities (in terms of the competitive effect on other individuals), as assumed in our competition‐asymmetry hypothesis. Indeed, the estimated variance values were highest for the between‐species component (non‐phylogenetic one: σ_*k*_^2^ = 0.133, Appendix S1: Table S1), while between‐study variation was little (σ_*u*_^2^ = 0.000), suggesting that responses to spatial arrangements are highly species‐specific. The heterogeneity in competitive ability likely reflects that plant species share some common resources (e.g., light and nitrate) and they differ in their abilities to acquire and convert them. In this sense, species more sufficient in utilizing limited resources could be stronger effecters that perform better in a random treatment.

### Effects of phylogenetic distance on the response ratio

**FIGURE 3:**
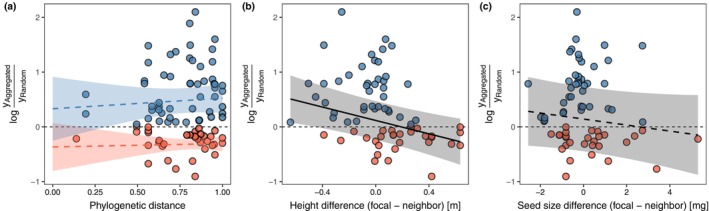
Relationships between the response ratio and (a) phylogenetic distance, and differences in (b) plant height and (c) seed size between focal and (multiple) competitor species. Each point corresponds to an observation, colored with blue and red when the response ratios were positive and negative, respectively. The lines and 95% CIs are drawn based on the estimates of the phylogenetic meta‐regressions (solid line: *p* < 0.05; broken line: *p* > 0.05).

Contrary to our prediction (Figure 1b,c), we observed no significant relationship between response ratios and (average) phylogenetic distances between a focal species and its competitor(s) (β_phylo,+_ = 0.193 ± 0.350 [SE], *p* = 0.581; β_phylo,–_ = 0.062 ± 0.267, *p* = 0.815, Figure 3a). Other studies have also concluded that phylogenetic distance between competitors may not be a good measure of their niche difference (Cahill et al., 2008; Germain et al., 2016; Godoy et al., 2014), and this can be attributed to several mechanisms. The most straightforward explanation would be that ecological traits underpinning niche differences are evolutionarily labile and therefore are not phylogenetically conserved (Silvertown et al., 2006). Secondly, if the relationship between niche difference and phylogenetic distance is asymptotic (Cavender‐Bares et al., 2009; Godoy et al., 2014), it would be undetectable when the studied species pairs are biased toward distant ones. This was the case in our dataset where combinations of close relatives were rare (Figure 3a), which might make it difficult to detect possible phylogenetic patterns if any (Cadotte et al., 2017). Also, we cannot dismiss the possibility that our analytical artifact—averaging the phylogenetic distance between a focal species and its multiple competitors—blurred the possible pattern.

### Effects of ecological dissimilarities on the response ratio

We found that response ratios were negatively correlated with differences in plant height between focal and competitor species (β_height_ = −0.613 ± 0.189, *p* = 0.001, Figure 3b). This indicates that larger species perform worse when conspecifics are aggregated, while smaller species do so when conspecific and heterospecific individuals are randomly arranged. For instance, some *Asteraceae* species—those demonstrating negative response ratios (Figure 2)—are reported to be highly productive (e.g., *Achillea millefolium* [Lamošová et al., 2010], *Grindelia camporum* [Porensky et al., 2012]). Hence, they likely impose strong competitive effects on other individuals (i.e., stronger affecters) via size‐dependent competition for light (Ferenc & Sheppard, 2020; Gaudet & Keddy, 1988). In contrast, some species with positive response ratios were much less dominant in the experiments (e.g., *Prunella vulgaris* [Lamošová et al., 2010], *Trifolium bifidum* [Porensky et al., 2012]). These species could experience reduced competition when surrounded by conspecific, weaker affecter individuals in the aggregation treatment. We found no significant relationship between response ratios and seed sizes (a trait sometimes considered as representing competitive ability), but it might be noteworthy that the estimated slope was negative as predicted (β_seed_ = −0.055 ± 0.042, *p* = 0.188, Figure 3c). Additionally, we found no evidence that other ecological traits (seed dispersal modes, functional types, and life history) of focal species were significantly associated with the response to spatial arrangements (Table 1).

**TABLE 1:** Summary statistics of the estimation of the meta‐regression that relates the response ratio to ecological traits (life history, functional types, and seed dispersal modes) of the focal species.

Coefficient	Estimate	95% CI	SE	*p*
Intercept	0.222	−0.592, 1.036	0.415	0.593
Life history: Perennial	−0.054	−0.378, 0.270	0.165	0.745
Functional type: Grass	−0.015	−1.474, 1.444	0.745	0.984
Functional type: Legume	−0.218	−1.481, 1.046	0.645	0.735
Seed dispersal: Wind	0.123	−0.213, 0.458	0.171	0.474
Seed dispersal: Others	0.432	−0.117, 0.981	0.280	0.123

*Note*: For these categorical variables, reference levels were set as annual (for life history), forb (functional type), and gravity (seed dispersal mode).

These results lend support to the competitive asymmetry hypothesis (Figure 1c). They align with previous empirical evidence that plant competitive abilities are linked to certain ecological traits (Ferenc & Sheppard, 2020; Gaudet & Keddy, 1988; Kraft et al., 2015; Pérez‐Ramos et al., 2019; Turnbull et al., 2004). However, although a non‐negligible number of observations (53 out of 89) demonstrating positive response ratios contrast with the competition‐related hypothesis (Figure 1b), we cannot rule out the possibility that interspecific niche differences partially influence species' responses to spatial arrangements, particularly for the cases with negative response ratios. This is because we could not consider other ecological traits that are believed to underpin niche differences in plants, such as root depth (Nobel, 1997) and resource uses (Ashton et al., 2010). It is possible that, at least for the species demonstrating negative response ratios (i.e., species experiencing stronger intraspecific competition), their response ratios may be explained by interspecific differences in such traits (Kraft et al., 2015; Pérez‐Ramos et al., 2019). The effects of absolute interspecific differences in plant height or seed size on response ratios, however, were nonsignificant (absolute plant height difference: coefficient = 0.151 ± 0.312, *p* = 0.628; absolute seed size difference: coefficient = −0.085 ± 0.048, *p* = 0.077, Appendix S1: Figure S2), indicating that these traits may not be responsible for interspecific niche differences.

## CONCLUSION

The finding that many species benefitted from spatial aggregation (Figure 2) suggests that interspecific spatial segregation may promote their coexistence at large spatial scales by rescuing competitively inferior species (Hart & Marshall, 2009; Stoll & Prati, 2001). Given that conspecific aggregation is ubiquitous in plant communities (Nishizawa et al., 2022), dispersal limitation could play a pivotal role in plant coexistence. Although we restricted ourselves to studying plant communities, this conclusion would be applicable to other ecosystems in which species exhibit conspecific aggregation. For example, numerous studies have demonstrated conspecific aggregation patterns in insects (Atkinson & Shorrocks, 1984; Inouye, 1999; Ohsaki et al., 2022) and sessile marine invertebrates (Karlson et al., 2007; Keough, 1983), and these species are known to respond idiosyncratically to aggregation (Fader & Juliano, 2013; Hart & Marshall, 2009; Idjadi & Karlson, 2007; Kadowaki & Inouye, 2015).

Furthermore, our results lend support for the competitive asymmetry hypothesis that interspecific differences in competitive ability (in terms of the competitive effect on other individuals) determine the response to spatial arrangements. This finding suggests that strong competition could lead to the dominance of stronger affecter species and underdispersion (clustering) of ecological traits within communities. Therefore, the traditional approach to link trait overdispersion within a community to the intensity of competition (e.g., Webb et al., 2002)—as predicted by the competition‐relatedness hypothesis—should be considered carefully (Mayfield & Levine, 2010).

## CONFLICT OF INTEREST STATEMENT

The authors declare no conflicts of interest.

## Supporting information


Appendix S1.

## Data Availability

Data and code (Shinohara, 2025) are available in Figshare at https://doi.org/10.6084/m9.figshare.25911682.v1.
