# Supplementary material for: Competitive ability underpins the effect of spatial aggregation on plant performance
Source: Ecology. 2025 Apr 10;106(4):e70075. doi: 10.1002/ecy.70075 (PMC11983673; doi:10.1002/ecy.70075)
Supplement: Supplementary file 1 — Appendix S1. [file ECY-106-e70075-s001.pdf]

Appendix S1

Title: Competitive ability underpins the effect of spatial aggregation on plant performance

Authors: Naoto Shinohara, Haruna Ohsaki

Journal: Ecology

Figure S1

Effects on the response ratio of experimental settings, namely (a) the duration of experiment (in month), (b) the density (m^{-2}) of planted individuals or sown seeds, and (c) the unit size of aggregation. The lines were drawn based on the meta-regressions separately fitted to the positive- and negative-response ratio data. Note that in panel (b), the regression models were separately fitted to data with different density units (i.e., the number of planted individuals or the sewn seeds). None of the estimated slopes were statistically significant ($p > 0.05$) except for the effect of the unit size of aggregation in the data with negative response ratios (the red line in panel (c), slope = –0.038, SE = 0.018, $p = 0.034$).

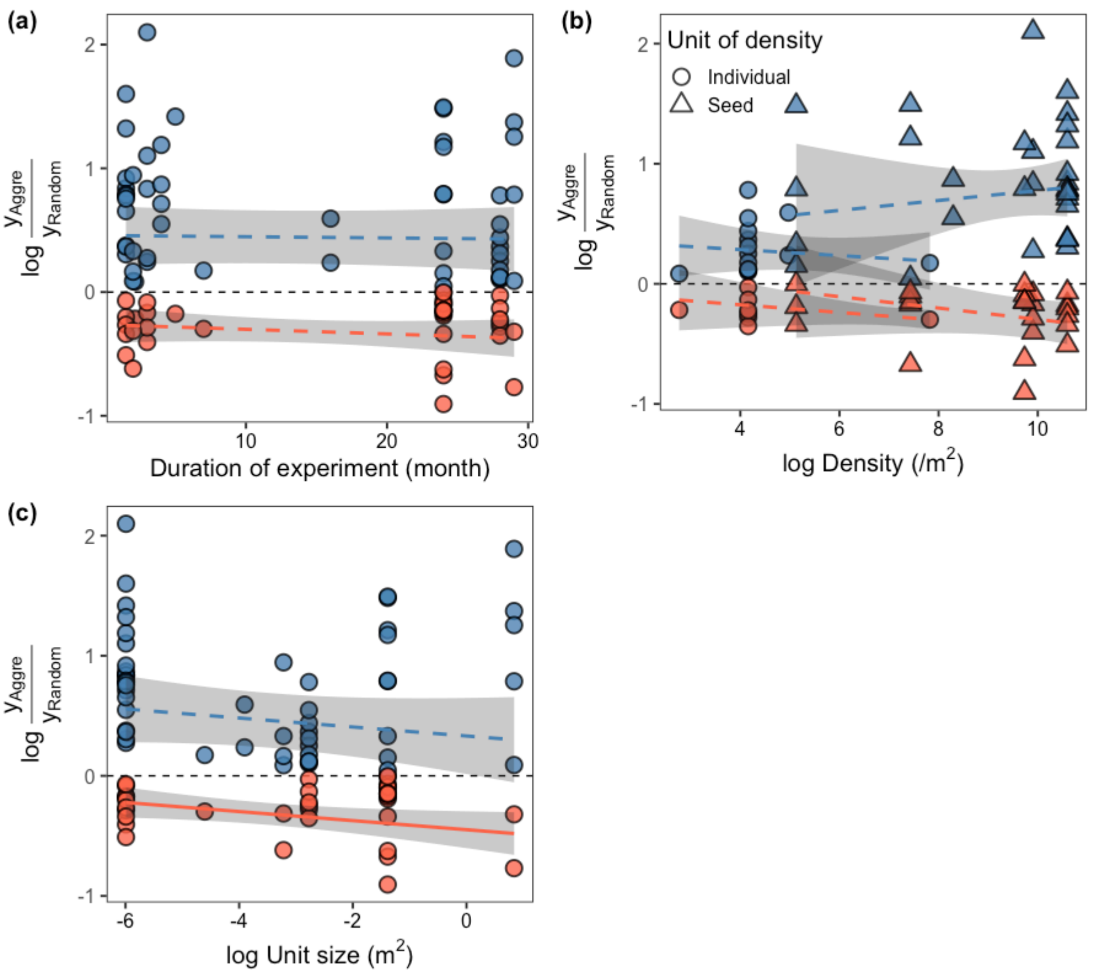

Figure S2

Relationships between the response ratio and *absolute* differences in (a) plant height and (b) seed size between focal and (multiple) competitor species. To be consistent with the prediction of the competition-relatedness hypothesis (see Fig. 1b in the main text), the data with negative response ratios were analyzed. The lines and 95% confidence intervals are drawn based on the estimates of the phylogenetic meta-regressions (p >0.05 for both panels).

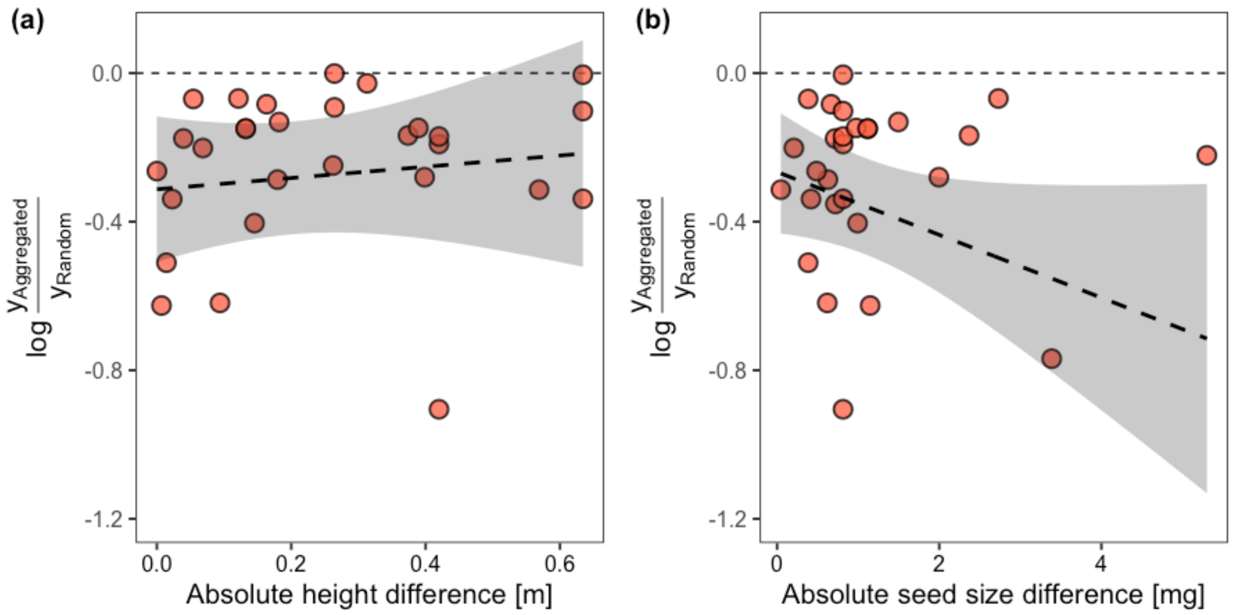

Table S1

Estimates of the random-effect variance components.

	Estimate
Among-study: σ_u^2	0.000
Non-phylogenetic among-species: σ_k^2	0.133
Phylogenetic among-species: σ_a^2	0.014

Table S2

Trait (dispersal mode, seed mass, and plant height) values of the species. The data were obtained from publicly available databases (Database of European Vegetation, Habitats and Flora [www.floraveg.eu], TRY [www.try-db.org], Plants of the World Online, and GBIF), or previous studies. The refered data sources are shown as well.

Species	Dispersal mode	Dispersal mode reference	Seed mass (mg)	Seed mass reference	Plant height (m)	Plant height reference
Achillea millefolium	NA	NA	0.07	Thompson et al., 2001	0.5	FloraVeg.eu
Alternanthera philoxeroides	gravity	Schmid et al., 2023	NA	NA	0.6	FloraVeg.eu
Amorpha canescens	NA	NA	2.26	Foster and Tilman, 2003	0.462	TRY
Andropogon gerardii	wind	McKone et al., 1998	2.4	FloraVeg.eu	0.79	TRY
Artemisia indica	wind	Shimono et al., 2013	NA	NA	NA	NA
Astragalus canadensis	gravity	https://www.gbif.org/ja/	NA	NA	0.995	TRY
Bolboschoenus planiculmis	gravity	Lososová et al., 2023	2.749	Lovas-Kiss et al., 2020	0.68	FloraVeg.eu

Bromus pectinatus	gravity	https://www.gbif.org/ja/	NA	NA	0.5	TRY
Calandrinia ciliata	gravity	Szojka, 2020	0.37	Gurney et al., 2015	NA	NA
Calendula arvensis	others	De Clavijo, 2005	4.84	FloraVeg.eu	0.22	FloraVeg.eu
Capsella bursa-pastoris	gravity	https://powo.science.kew.org/	0.0933	Orsucci et al., 2020	0.22	FloraVeg.eu
Cardamine hirsuta	gravity	Hay et al., 2014	0.09339754	Cao et al., 2016	0.14	FloraVeg.eu
Carex neurocarpa	gravity	Xue et al., 2018	NA	NA	NA	NA
Centaurea cyanus	gravity	Le Corre et al., 2014	1.48	Spencer and Liow, 2014	0.525	TRY
Coreopsis lanceolata	gravity	Marazzi et al., 2022	0.747	Folgate and Scheiner, 1992	0.29	FloraVeg.eu
Dalea purpurea	gravity	https://www.gbif.org/ja/	1.53	Foster and Tilman, 2003	0.356	TRY
Desmanthus illinoensis	gravity	Zorn-Arnold et al., 2006	0.61	Kulakow, 1999	1.20365	TRY
Desmodium canadense	gravity	Skogen et al., 2010	NA	NA	1.05	TRY
Echinacea angustifolia	gravity	Wagenius, 2004	4.11	Parsons et al., 2018	NA	NA

Elymus	gravity	https://www.gbif.org/ja/	NA	NA	NA	NA
Elymus canadensis	NA	NA	3.97	FloraVeg.eu	1.14	FloraVeg.eu
Elymus smithii	gravity	https://powo.science.kew.org/	NA	NA	0.53	TRY
Elymus trachycaulus	NA	NA	3.35	FloraVeg.eu	0.69	FloraVeg.eu
Erigeron annuus	wind	Trtikova et al., 2011	0.02558	Yong et al., 2015	0.86	FloraVeg.eu
Festuca elata	NA	NA	NA	NA	NA	NA
Festuca rubra	NA	NA	1.365	Fairey and Lefkovitch, 1996	0.28335	TRY
Fragaria vesca	others	https://powo.science.kew.org/	0.35	Fröborg, 2001	0.13	FloraVeg.eu
Glycyrrhiza lepidota	others	https://www.gbif.org/ja/	7	Diehl et al., 2017	NA	NA
Grindelia hirsutula	gravity	https://www.gbif.org/ja/	NA	NA	NA	NA
Helianthus maximiliani	gravity	https://www.gbif.org/ja/	NA	NA	1.061	TRY
Heliopsis helianthoides	gravity	Seahra et al., 2019	3.88	Foster and Tilman, 2003	0.93	FloraVeg.eu
Hemarthria compressa	gravity	Huang et al., 2008	2.0802	Wang et al., 2017	NA	NA

Holcus lanatus	wind	Beddows, 1961	0.39	FloraVeg.eu	0.53	FloraVeg.eu
Hydrocotyle vulgaris	NA	NA	0.43	FloraVeg.eu	0.15	FloraVeg.eu
Ipomoea batatas	gravity	Lososová et al., 2023	NA	NA	1.5	TRY
Lespedeza capitata	NA	NA	2.42	Foster and Tilman, 2003	0.8125	TRY
Medicago lupulina	others	Yan et al., 2009	1.73	FloraVeg.eu	0.23	FloraVeg.eu
Melica californica	gravity	https://www.gbif.org/ja/	1.819	Lulow et al., 2007	NA	NA
Melilotus officinalis	wind	Akhalkatsi et al., 1999	2.146	Bu et al., 2007	1	FloraVeg.eu
Monarda fistulosa	NA	NA	0.33	FloraVeg.eu	0.94	FloraVeg.eu
Nassella pulchra	gravity	https://www.gbif.org/ja/	4.83	Charles et al., 2022	NA	NA
Nassella viridula	NA	NA	2.8	Carta et al., 2017	NA	NA
Panicum virgatum	gravity	Kwit and Stewart, 2012	1.28	Brown and Fridley, 2003	0.484	TRY
Plantago lanceolata	gravity	https://powo.science.kew.org/	1.52	FloraVeg.eu	0.23	FloraVeg.eu
Poa annua	wind	Hutchinson and Seymour, 1982	0.3	Springer and Goldman, 2016	0.11162025	TRY

Prunella vulgaris	gravity	Winn, 1988	0.67	Barsukova and Astashenkov, 2018	0.15	FloraVeg.eu
Ratibida columnifera	gravity	https://www.gbif.org/ja/	0.77	FloraVeg.eu	0.94	FloraVeg.eu
Ratibida pinnata	NA	NA	0.61	FloraVeg.eu	1.4	FloraVeg.eu
Rudbeckia	NA	NA	NA	NA	NA	NA
Sanguisorba officinalis	gravity	https://doi.org/10.5073/JABFQ.2021.094.011	3.01	FloraVeg.eu	0.7	FloraVeg.eu
Schizachyrium scoparium	wind	Gustafson et al., 2012	1.3	Springer, 2005	0.4225	TRY
Silene flos-cuculi	gravity	Aavik et al., 2012	0.13	Zani and Müller, 2017	0.46	FloraVeg.eu
Solidago rigida	wind	Werner and Platt, 1976	0.49	Foster and Tilman, 2003	NA	NA
Sorghastrum nutans	wind	McKone et al., 1998	0.47	McKone et al., 1998	0.63916667	TRY
Stachys annua	gravity	Monzeglio and Stoll, 2008	1.04	FloraVeg.eu	0.21	FloraVeg.eu
Stellaria media	gravity	Briggs et al., 1991	0.58	Bu et al., 2007	0.18	FloraVeg.eu

Symphyotrichum novae-angliae	wind	https://www.gbif.org/ja/	0.43	FloraVeg.eu	1.03	FloraVeg.eu
Trifolium bifidum	gravity	Szojka, 2020	NA	NA	NA	NA
Trifolium willdenovii	NA	NA	2.14	Charles et al., 2022	NA	NA
Verbena hastata	gravity	https://www.gbif.org/ja/	0.2	Brown and Fridley, 2003	0.84	TRY

References

Aavik, T., Edwards, P., Holderegger, R., & Billeter, R. (2012). The potential genetic consequences of seed mixtures. ENHANCE: Enhancing ecosystem connectivity through intervention–benefits for nature and society.17–26.

Akhalkatsi, M., Pfauth, M. & Calvin, C.L. (1999). Structural aspects of ovule and seed development and nonrandom abortion inMelilotus officinalis (Fabaceae). *Protoplasma*, 208, 211−223.

Barsukova, I., & Astashenkov, A. (2018). Seed and biological productivity of Prunella vulgaris L. in situ and ex situ Irina Barsukova and Alexei Astashenkov BIO Web Conf., 11, 00004

Beddows, A. R. (1961), FLOWERING BEHAVIOUR, COMPATIBILITY AND MAJOR GENE DIFFERENCES IN HOLCUS LANATUS L. *New Phytologist*, 60, 312−324.

Briggs, D., Hodkinson, H. & Block, M. (1991). Precociously developing individuals in populations of chickweed [Stellaria media (L.) Vill.] from different habitat types, with special reference to the effects of weed control measures. *New Phytologist*, 117, 153−164.

Brown, R.L. & Fridley, J.D. (2003), Control of plant species diversity and community invasibility by species immigration: seed richness versus seed density. *Oikos*, 102, 15−24

Bu, H., Chen, X., Xu, X. et al. (2007) Seed mass and germination in an alpine meadow on the eastern Tsinghai−Tibet plateau. *Plant Ecology* 191, 127−149.

Cao, Y., Xiao, Y., Huang, H. et al. (2016) Simulated warming shifts the flowering phenology and sexual reproduction of Cardamine hirsuta under different Planting densities. *Scientific Reports*, 6, 27835.

Carta, A., Skourti, E., Mattana, E., Vandelook, F., & Thanos, C. A. (2017). Photoinhibition of seed germination: occurrence, ecology and phylogeny. *Seed Science Research*, 27, 131−153.

Charles, L. S., Maron, J. L., & Larios, L. (2022). Species provenance and traits mediate establishment and performance in an invaded grassland. *Functional Ecology*, 36, 1528−1541.

De Clavijo, E. R. (2005). The reproductive strategies of the heterocarpic annual Calendula arvensis (Asteraceae). *Acta Oecologica*, 28, 119−126.

Diehl, R. M., Merritt, D. M., Wilcox, A. C., & Scott, M. L. (2017) Applying Functional Traits to Ecogeomorphic Processes in Riparian Ecosystems. *BioScience*, 67, 729−743.

Fairey, N. A. & L. P. Lefkovitch. 1996. Crop density and seed production of creeping red fescue (Festuca rubra L. var. rubra). 2. Reproductive components and seed characteristics. *Canadian Journal of Plant Science*, 76, 299−306.

Folgate, L. A., & Scheiner, S. M. (1992). Distribution of a Restricted Locally Abundant Species: Effects of Competition and Nutrients on Coreopsis lanceolata. *The American Midland Naturalist*, 128, 254−269.

Foster, B. L. & Tilman, D. (2003). Seed limitation and the regulation of community structure in oak savanna grassland. *Journal of Ecology*, 91, 999−1007.

Fröborg, H. (2001). Seed size and seedling emergence in 16 temperate forest herbs and one dwarf-shrub. *Nordic Journal of Botany*, 21, 373−384.

Gurney, C. M., Prugh, L. R., & Brashares, J. S. (2015). Restoration of Native Plants Is Reduced by Rodent-Caused Soil Disturbance and Seed Removal. *Rangeland Ecology & Management*, 68, 359–366.

Gustafson, D. J., Harris-Shultz, K., Gustafson, P. E., Giencke, L. M., Denhof, R. C., & Kirkman, L. K. (2018). Seed Sourcing for Longleaf Pine Herbaceous Understory Restoration: Little Bluestem (Schizachyrium scoparium) and Hairy Lespedeza (Lespedeza hirta) Restoration Genetics. *Natural Areas Journal*, 38, 380−392.

Hay, A.S., Pieper, B., Cooke, E., Mand_kov_, T., Cartolano, M., Tattersall, A.D., Ioio, R.D., McGowan, S.J., Barkoulas, M., Galinha, C., Rast, M.I., Hofhuis, H., Then, C., Plieske, J., Ganal, M., Mott, R., Martinez-Garcia, J.F., Carine, M.A., Scotland, R.W., Gan, X., Filatov, D.A., Lysak, M.A. & Tsiantis, M. (2014). Cardamine hirsuta: a versatile genetic system for comparative studies. *the plant journal*, 78, 1−15.

Huang, L.-K., Zhang, X.-Q., Ma, X., Liu, W., Li, F. & Zeng, B. (2008), Genetic differentiation among Hemarthria compressa populations in south China and its genetic relationship with H. japonica. *Hereditas*, 145, 84−91.

Hutchinson, C. S., & G. B. Seymour. (1982). Poa Annua L. *Journal of Ecology* 70, 887−901.

Skogen, K. A., Senack, L., & Holsinger, K. E. (2010) Dormancy, small seed size and low germination rates contribute to low recruitment in Desmodium cuspidatum (Fabaceae). *The Journal of the Torrey Botanical Society*, 137, 355−365.

Kulakow, P.A. (1999) Variation in Illinois bundleflower (Desmanthus illinoensis (Michaux) MacMillan): A potential perennial grain legume. *Euphytica*, 110, 7−20.

Kwit, C. & Stewart, C. N. (2012), Gene flow matters in switchgrass (Panicum virgatum L.), a potential widespread biofuel feedstock. *Ecological Applications*, 22, 3−7.

Le Corre, V., Bellanger, S., Guillemin, J. P., & Darmency, H. (2014). Genetic diversity of the declining arable plant C entaurea cyanus: population fragmentation within an agricultural landscape is not associated with enhanced spatial genetic structure. *Weed research*, 54, 436−444.

Lososová, Z., Axmanová, I., Chytrý, M., Midolo, G., Abdulhak, S., Karger, D. N., Renaud, J., Van Es, J., Vittoz, P., & Thuiller, W. (2023). Seed dispersal distance classes and dispersal modes for the European flora. *Global Ecology and Biogeography*, 32, 1485–1494.

Lovas-Kiss Á, Vincze O, Kleyheeg E, et al. (2020). Seed mass, hardness, and phylogeny explain the potential for endozoochory by granivorous waterbirds. *Ecology and Evolution*. 10, 1413–1424.

Lulow, M. E., Young, T. P, Wirka, J. L., & Anderson, J. H. (2007). Variation in the Initial Success of Seeded Native Bunchgrasses in the Rangeland Foothills of Yolo County, California. *Ecological Restoration*, 25, 20−28.

Marazzi, B., Mangili, S., Andreas, G., & Jousson, A. (2022). Biology and spread of the new alien species Coreopsis grandiflora (Asteraceae) in southern Switzerland. *Bollettino della Società ticinese di scienze naturali*, 110, 57–70.

Orsucci M., Milesi P., Hansen J., Girodolle J., Glémin S. & Lascoux M. (2020). Shift in ecological strategy helps marginal populations of shepherd's purse (Capsella bursa-pastoris) to overcome a high genetic load. *Proceedings of the Royal Society B*, 287: 20200463.

Parsons, J. L., Liu, R., Smith, M. L., & Harris, C. S. (2018). Echinacea fruit: phytochemical localization and germination in four species of Echinacea. *Botany*, 96, 461−470.

McKone, M. J., Lund, C. P. & O'Brien, J. M. (1998). Reproductive biology of two dominant prairie grasses (Andropogon gerardii and sorghastrum nutans, Poaceae): male-biased sex allocation in wind-pollinated plants?. *American Journal of Botany,* 85, 776−783.

Monzeglio, U. & Stoll, P. (2008). Effects of spatial pattern and relatedness in an experimental plant community. *Evolutionary Ecology*, 22, 723−741.

Schmid, S.A., Turnage, G. & Ervin, G.N. (2023). Rare Production of Seeds by Invasive Alternanthera philoxeroides (Alligator Weed) in North America Observed in Terrestrial Populations. *Wetlands*, 43, 12.

Seahra, S., Yurkonis, K.A. & Newman, J.A. (2019). Seeding tallgrass prairie in monospecific patches promotes native species establishment and cover. *Restoration Ecology*, 27, 82−91.

Shimono Y., Hayakawa, H., Kurokawa, S., Nishida, T., Ikeda, H. & Futagami, N. (2013). Phylogeography of Mugwort (Artemisia indica), a Native Pioneer Herb in Japan. J*ournal of Heredity*, 104, 830−841.

Springer, T.L. (2005). Germination and Early Seedling Growth of Chaffy-Seeded Grasses at Negative Water Potentials. *Crop Science*, 45, 2075−2080.

Springer, T. & Goldman, J. (2016). Seed Germination of Five Poa Species at Negative Water Potentials. *American Journal of Plant Sciences*, 7, 601−611.

Spencer, D. F. & Liow P-S. (2014) Variation In Seed Characteristics And Growth For Thistles (Cardueae: Asteraceae) In California And Oregon, *Madroño*, 61, 339−349.

Szojka, M. (2020). Persistence across habitat boundaries : revealing the demographic fates of failed dispersers in patchy landscapes. Electronic Theses and Dissertations (ETDs) 2008+. University of British Columbia.

Thompson, K, Jalili A, Hodgson JG, et al. (2001). Seed size, shape and persistence in the soil in an Iranian flora. *Seed Science Research*. 11, 345−355.

Trtikova, M., Güsewell, S., Baltisberger, M. & Edwards, P. J. (2011). Distribution, growth performance and genetic variation of Erigeron annuus in the Swiss Alps. *Biological Invasions*, 13, 413−422.

Wagenius, S. (2004). Style Persistence, Pollen Limitation, and Seed Set in the Common Prairie Plant Echinacea angustifolia (Asteraceae). *International Journal of Plant Sciences*, 165, 595−603.

Werner, P. A., & Platt, W. J. (1976). Ecological Relationships of Co-Occurring Goldenrods (Solidago: Compositae). *The American Naturalist*, 110, 959−971.

Wang, Z., Qian, J., & Liu, B. (2017). Seed Mass, Shape and Dormancy in Arid Temperate Degraded Grassland in Northeastern Inner Mongolia, China. *Land Degradation & Development*, 28, 887−896.

Winn, A. A. (1988), Ecological and Evolutionary Consequences of Seed Size in Prunella Vulgaris. *Ecology*, 69, 1537−1544.

Yong, XH., Liu, JH., Li, Z., Du, SF., Zhang, ZW., Meng, XF., Wu, XJ. & Wang, YJ. (2015). Maternal Mowing Effect on Seed Traits of an Invasive Weed, Erigeron annuus in Farmland. *Sains Malaysiana*. 44, 347-354.

Xue, W., Huang, L., Yu, FH. & Bezemer, T. M. (2018). Intraspecific aggregation and soil heterogeneity: competitive interactions of two clonal plants with contrasting spatial architecture. *Plant and Soil* 425, 231−240.

Yan, J., Chu, H. J., Wang, H. C., Li, J. Q., & Sang, T. (2009). Population genetic structure of two Medicago species shaped by distinct life form, mating system and seed dispersal. *Annals of botany*, 103, 825−834.

Zani, D. & Müller, J. V. (2017). Climatic control of seed longevity of Silene during the post-zygotic phase: do seeds from warm, dry climates possess higher maturity and desiccation tolerance than seeds from cold, wet climates?. *Ecological Research*, 32, 983−994.

Zorn-Arnold, B., Brown, J. S., & Howe, H. F. (2006). Obvious and cryptic vole suppression of a prairie legume in experimental restorations. *International Journal of Plant Sciences*, 167, 961−968.
